# Magnetic Fe_3_O_4_@BTC nanocomposite for ultrasound-assisted synthesis of dihydropyrano[2,3-*c*]pyrazoles

**DOI:** 10.1039/d5ra08120c

**Published:** 2025-12-12

**Authors:** Santosh A. Fuse, Somnath C. Dhawale, Balaji B. Mulik, Raviraj P. Dighole, Balaji R. Madje, Bhaskar R. Sathe

**Affiliations:** a Department of Chemistry, Dr Babasaheb Ambedkar Marathwada University Chhatrapati Sambhajinagar Maharashtra 431004 India bsathe.chemistry@bamu.ac.in; b Vasantrao Naik College Chhatrapati Sambhajinagar Maharashtra 431003 India drmadjebr@gmail.com; c A. S. C. College, Badnapur Dist.-Jalna Maharashtra 431202 India; d MGM University Chhatrapati Sambhajinagar Maharashtra 431001 India; e Department of Nanoscience and Technology, Dr Babasaheb Ambedkar Marathwada University Chhatrapati Sambhajinagar Maharashtra 431004 India

## Abstract

We report an efficient approach for the synthesis of medicinally important dihydropyrano[2,3-*c*]pyrazoles derivatives by using Fe_3_O_4_@BTC nanocomposite (NCs) based catalytic system. The Fe_3_O_4_@BTC NCs were successfully synthesised *via*. immobilizing benzene-1,3,5-tricarboxylic acid (BTC) on Fe_3_O_4_ magnetic nanoparticles (MNPs). The synthesised NCs were characterized using X-ray diffraction (XRD) which disclose the formation of a crystalline structure of Fe_3_O_4_@BTC NCs, Fourier transform infrared (FTIR) spectroscopy suggests the presence of Fe–O band at 576 cm^−1^ in addition to –C=O, –O–H stretching frequencies are also observed, field emission scanning electron microscopy (FE-SEM) represents the spherical shape of Fe_3_O_4_@BTC NCs, high resolution-transmission electron microscopy (HR-TEM) revealed the particle size tobe ∼10.335 nm, energy dispersive analysis of X-ray (EDAX) shows the presence of Fe, C and O elements, Brunauer–Emmett–Teller (BET) surface area reveals its specific surface area 84.87 m^2^ g^−1^ and thermogravimetric analysis (TGA) shows its exceptional higher thermal stability. Furthermore, all dihydropyrano[2,3-*c*]pyrazoles derivatives were synthesised with high yield (79–92%), in shorter time (4–12 min). Recyclability of NCs was also investigated and it was found that the NCs can be reused over five cycles without any significant loss in its activity. Significantly, this protocol has prominent benefits such as high yields of product, shorter reaction time, facile workup, recyclable, use of ultrasound as clean energy source and absence of toxic solvents.

## Abstract 2

An efficient approach for the synthesis of medicinally important dihydropyrano[2,3-*c*]pyrazoles derivatives by using Fe_3_O_4_@BTC nanocomposite (NCs) based catalytic system.

## Graphical abstract

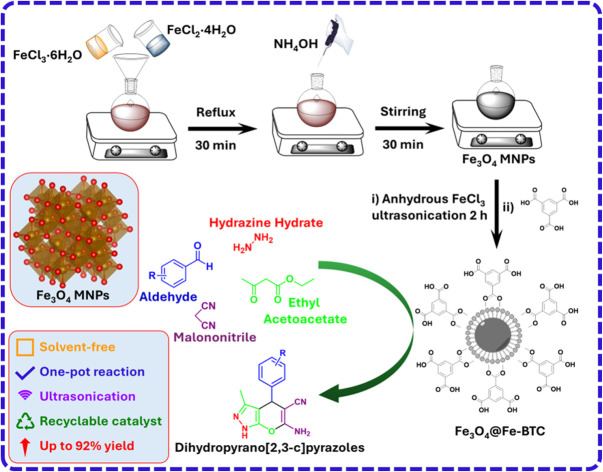

## Introduction

**Scheme 1:**
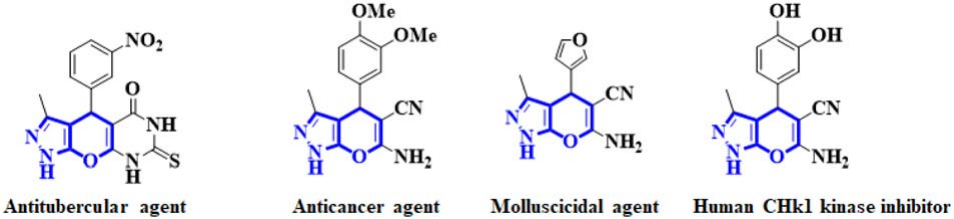
Selected examples of dihydropyrano[2,3-*c*]pyrazoles derivatives with biological and pharmacological activities.

Heterocycles are a building block of proteins, amino acids, chlorophyll and haemoglobin hence essential to living things.^1–3^ Dihydropyrano[2,3-*c*]pyrazole core have attracted considerable attention over the recent years because of their promising biological and pharmacological activities such as phosphodiesterase (PDE) inhibitors,^4^ anti-HIV,^5^ anti-inflammatory,^6^ anti-microbial,^7^ anti-cancer,^8^ anti-fungal,^9^ and anti-oxidant^10^ properties and selected examples are shown in Scheme 1.

Due to versatility of dihydropyrano[2,3-*c*]pyrazole scaffolds various methods were reported for its synthesis. Substantially, the multicomponent reaction (MCRs) of aromatic aldehyde, malononitrile, ethyl acetoacetate and hydrazine hydrate, which provides dihydropyrano[2,3-*c*]pyrazole as products is one of the accepted MCRs.^11^ In recent years, MCRs are eco-friendly synthetic strategy as they offer unique advantages such as efficient, atom economic, minimization of waste and time saving.^12^

In this regards, solvent free synthesis of dihydropyrano[2,3-*c*]pyrazoles have been organized as practical synthetic approach emphasizing operational simplicity, reducing hazardous chemical solvents and enhances product selectivity.^13^ Further, such reactions are performed under environment friendly ultrasound waves without using conventional energy sources.^14^ Ultrasound irradiation is useful technique used in the organic synthesis of various medicinal and biological active compounds. Under ultrasound irradiation organic transformation occurs in high yield, short reaction time and greater selectivity.^15,16^

Nowadays, magnetic NCs are considered as ecologically and economically sound alternatives to traditional catalyst which frequently display high specific surface area to volume ratio.^17^ Sometime NCs cannot be used directly as they are connected with a few restrictions. These issues can be frequently overcomes with surface alteration with different layers. In that sense, surface coated magnetic NCs have attracted great attention.

They have remarkable physical and chemical properties such as thermal stability, low toxicity, ease of functionalization, high surface area, and effortless separations by an external magnet from reaction medium.^18,19^

As metal NCs with useful metals exhibits superior activity due to more expose active sites. Baoyu Wang *et al.* synthesised dual-size heterogeneous N-doped cobalt catalysts utilized in synthesis bio-based benzimidazoles.^20^ Jie Li *et al.* reported photo assisted dual catalytic systems to build various N containing organic molecules.^21^

Already shows their efficacy in the catalysis for the synthesis of variety of organic molecules^22^ Additionally, Fe based catalysts useful in area of energy like fuel cell, water splitting *etc.*^23,24^ In recent years, numerous Fe based MNP_S_ have been used for the synthesis of pyranopyrazoles derivatives under different conditions.^25,26^ The synthesis of dihydropyrano[2,3-*c*]pyrazoles derivatives is the core of many synthetic routes for drug synthesis. Some of these developing catalyst includes, Fe_3_O_4_@THAM-SO_3_H in which sulfuric acid coupling as an acidic group on magnetic core.^27^ In Fe_3_O_4_@chitsosan–tannic acid protocol chitosan decorated which has –NH_2_ and –OH groups provide platform for further modification while tannic acid offers acidity.^28^ Elhamifarm *et al.* synthesised YS-Fe_3_O_4_@PMO/IL-Cu in which magnetic mesoporous organosilica were linked with Cu-complex with ionic liquids linker.^29^ Almashhadani *et al.* has synthesise novel Cu based Schiff base complex with *O*-phenylenediamine supported by Fe_3_O_4_ magnetic core employed for pyrano[2,3-*c*]pyrazole heterocycles.^30^ In Fe_3_O_4_@PDA/CuCl_2_ synthesis, magnetite dopamine is decorated with Cu nanoparticles (Lewis's acid).^31^ For the synthesis of Fe_3_O_4_@THAM-piperazine, Fe_3_O_4_ MNPs coated with THAM (tris(hydroxymethyl)aminomethane) followed by piperazine immobilization.^32^ Ghasemzadeh *et al.* synthesised eco-friendly and non-toxic Fe_3_O_4_@l-arginine nanocatalyst for the synthesis of pyranopyrazoles derivatives.^33^ In biocompatible core/shell Fe_3_O_4_@NFC@Co(ii) catalyst effectively synthesises of pyranopyrazoles derivatives.^34^ Recently, Gholtash *et al.* has fabricated the Fe_3_O_4_ MNPs based on the immobilization of tungstic acid onto 3-chloropropyl-grafted TiO_2_ in enhancing its catalytic performance towards effective synthesis of pyrano[2,3-*c*]pyrazole derivatives.^35^ Behrouz Eftekhari far *et al.* make a use of nanobentonite (NB) surface, over developed with Fe_3_O_4_, organic linkers and sulfonic acid as NB-Fe_3_O_4_@SiO_2_@CPTMO@DEA-SO_3_H catalyst.^36^ However, some of communicated protocols are associated with some limitations such as using toxic solvents, multistep synthesis, long reaction time, harsh reaction conditions and higher cost of catalyst.^37,38^ In consideration of these weaknesses, ongoing research has been directed toward developing of new efficient catalytic system to synthesising significant scaffolds.

In accordance with reported literature Fe_3_O_4_ is demonstrated as to be an excellent surface where we can decorate different organic compounds with metals such as BTC (benzene-1,3,5-tricarboxylic acid), alginate, MCM-41, Cu, CuO, Sn(ii), Fe and As(iii)*etc.*^39–42^ In recent years, various Fe_3_O_4_ surface tailored with BTC NCs, received considerable applications in various fields such as hydration of nitriles, photocatalyst, esterification, environmental remediation, wastewater purification, and solid phase extraction.^43–45^ Niusha Nikooei *et al.* successfully decorated benzene-1,3,5-tricarboxylic acid on the MCM-41 surface and then utilized in the synthesis of 2,3-dihydroquinazolin-4(1*H*)-ones *via* one-pot three-component reaction.^46^ To the best of our knowledge the hybrid nanocomposite of Fe_3_O_4_ and BTC were never tried for dihydropyrano[2,3-*c*]pyrazoles.

Considering the importance of benzene-1,3,5-tricarboxylic acid (BTC) and scope of their applications in synthesizing metal–organic framework which motivates researchers.^47,48^ The BTC have received significant consideration due to their carboxylic acid functional groups attached at 1, 3 and 5 carbon atoms and extensively used as a linker in the synthesis of variety of nanocatalyst. Koosha *et al.* utilizes BTC in the synthesis of Pd NPs crosslinked with sodium alginate for oxidative amidation of organic moieties.^49^ Oveisi *et al.* synthesised bisnaphthols and of quinazolin-4(3*H*)-ones with Fe(BTC) as an iron-based metal–organic framework.^50^ This structure assists easy surface modifications of Fe_3_O_4_ MNPs and immobilizations of metals on it.

In this work, NCs consisting of Fe_3_O_4_ MNPs and modified its surface with BTC introduced. Our purpose is to take benefit from both properties Fe_3_O_4_ MNPs and BTC to develop efficient catalyst. The modified Fe_3_O_4_@BTC magnetic NCs then utilized in synthesis of dihydropyran[2,3-*c*]pyrazoles derivatives.

**Scheme 2:**
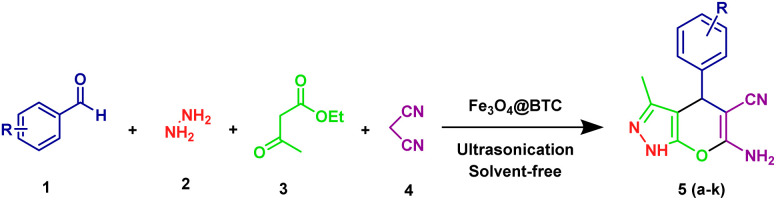
Synthesis of dihydropyrano[2,3-*c*]pyrazoles derivatives.

We reports an environmentally benign and efficient method for synthesis of dihydropyrano[2,3-*c*]pyrazoles derivatives under ultrasonic irradiation, solvent free environment using the Fe_3_O_4_@BTC as an eco-friendly NCs (Scheme 2).

## Experimental section

### Material and methods

All organic solvents and reagents including ammonium hydroxide (NH_4_·OH), ferric chloride hexahydrate (FeCl_3_·6H_2_O), ferrous chloride tetrahydrate (FeCl_2_·4H_2_O), benzene 1,3,5-tricarboxylic acid (BTC), anhydrous FeCl_3_, ethyl alcohol (C_2_H_5_OH), methyl alcohol (CH_3_OH) and dicholoromethane (DCM) were procured from commercial sources (Sigma-Aldrich and Loba Chemie). No further purification was performed on organic solvents and reagents and used without further purifications. Fourier-transform infrared (FT-IR) spectras were recorded by PerkinElmer instrument within the range of 400 to 4000 cm^−1^. The filed emission scanning electron microscopy (FE-SEM) images were recorded with JEOL-JSM7610F PLUS model. The high resolution transmission electron microscopy (HR-TEM) and energy dispersive analysis of X-ray (EDAX) studies were recorded with Model JEOL JEM 2100 PLUS instrument. The crystal structure pattern of material were examined through X-ray powder diffraction (XRD) using PANalytical X'Pert PRO diffractometer. The thermal stability of material was confirmed with thermogravimetric analysis (TGA) using PerkinElmer (STA) 8000 instrument. Brunauer–Emmett–Teller (BET) was measured by Quantachrome Novae 2200 instrument. Open capillary method was used to melting point measurement. The reaction progress was look over with thin layer chromatography (TLC) has been carry out on Silica gel 60F_254_ plates. All compounds were characterized using ^1^H-NMR and ^13^C-NMR and spectra were recorded on Bruker Advanced Neo (500 MHz and 400 MHz) spectrometer using DMSO as a solvent. Electrospray ionization mass spectra (ESI-MS) were recorded on Micromass Quattro micro instrument.

### Synthesis of Fe_3_O_4_ magnetic nanoparticles

The Fe_3_O_4_ MNPs has been synthesised in accordance with literature.^51^ Initially ferric chloride hexahydrate (FeCl_3_·6H_2_O) (2 mmol) and ferrous chloride tetra hydrate (FeCl_2_·4H_2_O) (1 mmol) was dissolved in a 100 mL of deionized water and this solution was refluxed for about 30 min followed by cool down to room temperature. Thereafter, 10 mL of 25% ammonia was added dropwise which resulted in the formation of black Fe_3_O_4_ precipitate. Then it was kept under strong and constant stirring for 30 min at room temperature. Finally, resulting Fe_3_O_4_ nanoparticles were separated using an external magnet rinsed with ethanol three times until its pH comes neutral. Then after Fe_3_O_4_ nanoparticles were dried in an oven at 80 °C.

### Synthesis of Fe_3_O_4_@BTC NCs

**Scheme 3:**
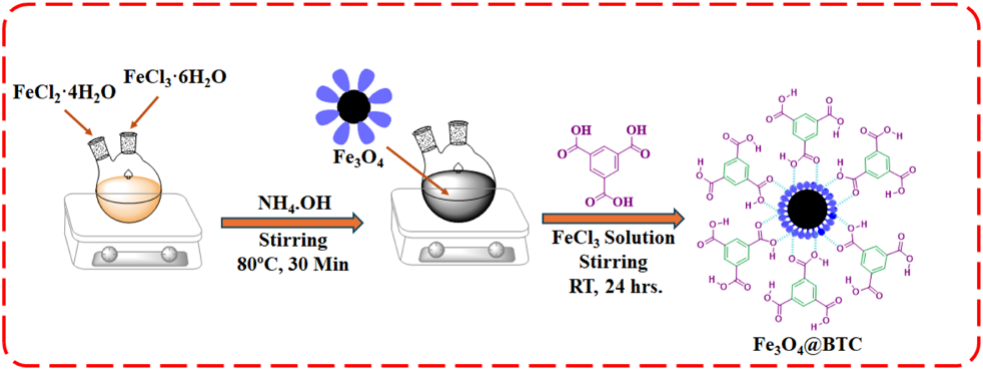
Schematic representation of synthesis of Fe_3_O_4_@BTC NCs.

The catalyst Fe_3_O_4_@BTC NCs has been prepared according to literature.^45^ Dried Fe_3_O_4_ (1 g) MNPs were dispersed in 50 mL of an ethanol solution of FeCl_3_ (50 mM) and kept for ultrasonication for 2 h. Next, 50 mL solution of benzene 1,3,5-tricarboxylic acid (10 mM) in ethanol was mixed to the reaction mixture by dropwise addition. Then it kept for mechanical stirring for 24 h at room temperature. Finally, Fe_3_O_4_@BTC NCs was separated with the help an external magnet, rinsed with ethanol three times and dried in an oven for at 80 °C (Scheme 3).

### General procedure for the synthesis of dihydropyrano[2,3-*c*]pyrazoles derivatives

A mixture of substituted aromatic aldehyde (1 mmol), malononitrile (1 mmol), ethyl acetoacetate (1 mmol), hydrazine hydrate (1 mmol) and Fe_3_O_4_@BTC (0.04 g) was taken in a 100 mL round bottom flask. The mixture was sonicated for 4–20 min. The reaction progress was monitored by TLC. After completion of reaction, reaction mixture was dissolved in ethanol. Then catalyst was separated from reaction mixture by applying external magnet. The resulting crude product was then purified by recrystallization. The separated Fe_3_O_4_@BTC NCs was then washed with ethanol to extract the adsorbed organic material and dried in oven.

### Spectroscopic data of representative compounds

#### (5a): 6-Amino-1,4-dihydro-3-methyl-4-phenylpyrano[2,3-*c*]pyrazole-5-carbonitrile

FT-IR (KBr, cm^−1^); 3423, 3166, 3005, 2185, 1708, 1645, 1402, 1037; ^1^H NMR (400 MHz, DMSO): *δ* 12.09 (s, 1H), 7.82–7.09 (m, 5H), 6.82 (d, 2H), 4.58 (s, 1H), 1.77 (s, 3H); ^13^C NMR (101 MHz, DMSO); *δ* 160.89, 154.76, 144.47, 135.60, 128.46, 127.49, 126.76, 120.84, 97.66, 57.17, 36.25, 9.56; MW: 252.27, observed MW 249.52.

#### (5e): 6-Amino-4-(2-chlorophenyl)-3-methyl-1,4-dihydropyrano[2,3-*c*] pyrazole-5-carbonitrile

FT-IR (KBr, cm^−1^); 3390, 3065, 2189, 1701, 1653, 1489, 1030, ^1^H NMR (400 MHz, DMSO); *δ* 12.13 (s, 1H), 7.44–7.15 (m, 4H), 6.95 (s, 2H), 5.06 (s, 1H), 1.76 (s, 3H), ^13^C NMR (101 MHz, DMSO); *δ* 161.59, 154.97, 140.97, 135.43, 132.00, 130.75, 129.52, 128.62, 127.79, 120.48, 96.89, 56.15, 33.90, 10.22.

#### (5j):6-Amino-4-(4-methoxyphenyl)-3-methyl-1,4-dihydropyrano[2,3-*c*]pyrazole-5-carbonitrile

FT-IR (KBr, cm^−1^); 3240, 3114, 2344, 1684, 1593, 1438, 1170, 1028, ^1^H NMR (500 MHz, DMSO); *δ* 12.06 (s, 1H), 7.07 (d, 2H), 6.86 (d, 2H), 6.79 (s, 2H), 4.53 (s, 1H), 3.72 (s, 3H), 1.77 (s, 3H), ^13^C NMR (126 MHz, DMSO); *δ* 160.77, 158.08, 154.78, 136.51, 135.60, 128.52, 121.00, 114.01, 97.91, 57.75, 55.03, 35.46, 9.49.

## Result and discussion

### X-ray diffraction (XRD) analysis

**Fig. 1:**
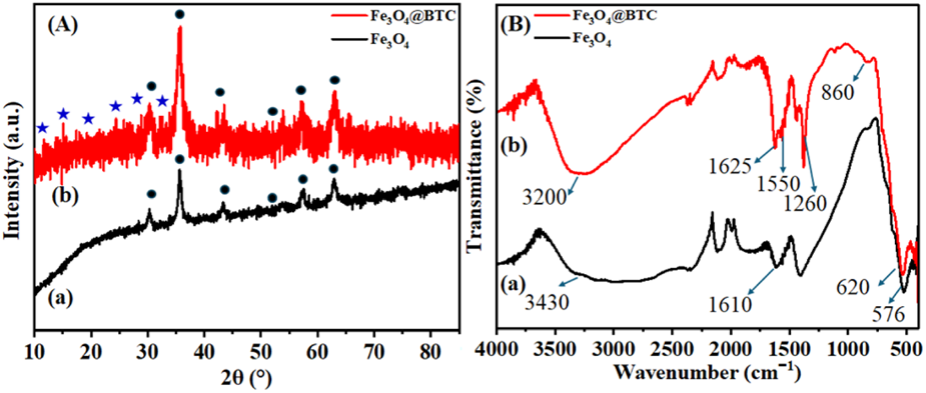
(a) Superimposed XRD pattern of Fe_3_O_4_ and Fe_3_O_4_@BTC NCs, (b) superimposed FT-IR spectrum of Fe_3_O_4_ and Fe_3_O_4_@BTC NCs.

X-ray diffraction (XRD) is important analysis technique employed for identification of crystalline material. The XRD pattern of Fe_3_O_4_ and Fe_3_O_4_@BTC NCs are presented in Fig. 1A. The diffraction pattern of Fe_3_O_4_ (Fig. 1A(a)) exhibits six diffraction peaks at 2*θ* = 30.1°, 35.6°, 43.2°, 53.6°, 57.2° and 62.8° (JCPDS card no. 19-0629) indexed as (220), (311), (400), (422), (511) and (440) reflections, correlates with the crystalline structure.^52^ In addition, to above mentioned peaks the extra peaks at 2*θ* = 10.6°, 14.2°, 18.3°, 23.4°, 27.5° and 32.1° appeared symbolizes the formation of Fe_3_O_4_@BTC NCs (Fig. 1A(b)).^53^

### Fourier transform infrared (FT-IR) analysis

**Fig. 2:**
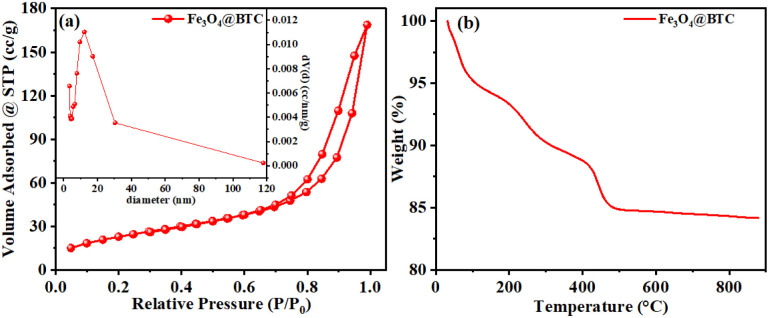
(a) N_2_-adsorption–desorption isotherms of Fe_3_O_4_@BTC NC, inset of (a) BJH curve of Fe_3_O_4_@BTC NCs (b) TGA profile of synthesised Fe_3_O_4_@BTC NC in air.

Fourier transform infrared (FT-IR) spectroscopy was used for identification of different functional groups present in Fe_3_O_4_ and Fe_3_O_4_@BTC NCs. The FT-IR spectrum of Fe_3_O_4_ and Fe_3_O_4_@BTC NCs was presented in Fig. 1B. In the spectrum of Fe_3_O_4_ (Fig. 1B(a)), absorption band were observed at 576 cm^−1^ and 620 cm^−1^ corresponding to Fe–O bond of crystalline lattice of Fe_3_O_4_ NCs. The stretching vibrations come out from surface –OH functional groups were noticed at 3430 cm^−1^ and 1610 cm^−1^ correlated with broad absorption band of –OH and bending vibration peak of –OH respectively.^54^ Further the covering of BTC over the Fe_3_O_4_ surface can be confirmed with the FT-IR spectrum of Fe_3_O_4_@BTC NCs (Fig. 2B(b)). The presence characteristic peak at 3200 cm^−1^ and 1625 cm^−1^ are correlates to –OH and C=O stretching band of carboxylic acid functionality respectively, as shown in (Fig. 2B(b)). The decrease in C=O frequency has been observed from 1700 cm^−1^ to 1625 cm^−1^ in NCs pointed the co-ordinating Fe with carboxylate group.^55,56^ Also, the signals were observed at 1550 cm^−1^ and 860 cm^−1^ are assigned to the –C=C– benzene ring stretching and –C–H benzene ring out of plane bending vibrations respectively. Additional stretching frequencies at 1260 cm^−1^ corresponds to –C–O bonds respectively,^57^ which indicates the formation of the Fe_3_O_4_@BTC NCs.

### N_2_-adsorption–desorption isotherms

The N_2_ adsorption desorption method is very important method to determine specific surface area of a NCs. Along with specific surface area pore diameter and pore volume of the NCs were also determined by BET and BJH methods. N_2_-adsorption–desorption isotherms were recorded at 77.35 k and presented in Fig. 2a. The slow adsorption was noticed in the *P*/*P*_0_ range of 0.0–0.2, afterwards fast increase in the *P*/*P*_0_ range of 0.2–0.1. The BJH pore volume and BET specific surface area of the Fe_3_O_4_@BTC NCs were 0.011 cm^2^ g^−1^ and 84.87 m^2^ g^−1^ (Fig. 2a) respectively. This result shows high porosity of Fe_3_O_4_@BTC NCs.

### Thermogravimetric (TGA) analysis

Thermal properties of the synthesised NCs were analysed using thermogravimetric (TGA) analysis in air atmosphere. This measures change in mass as a function of temperature and time, gives percentage loss of organic layers chemisorbed on the NCs surface. The TGA analysis curve of Fe_3_O_4_@BTC is included in Fig. 2b. The first weight loss (%) which observed below 150 °C can be assigned to removal of absorbed water and organic solvents on the surface of synthesised NCs. Furthermore, second weight loss was occurred in the range of 150–400 °C which can be attributed to the decomposition of organic layers like BTC on the surface of Fe_3_O_4_.^58^ Results indicate that BTC successfully stabilized on the Fe_3_O_4_ surface and offers thermal stability prior to 400 °C.

### Field emission scanning electron microscopic (FE-SEM) analysis

**Fig. 3:**
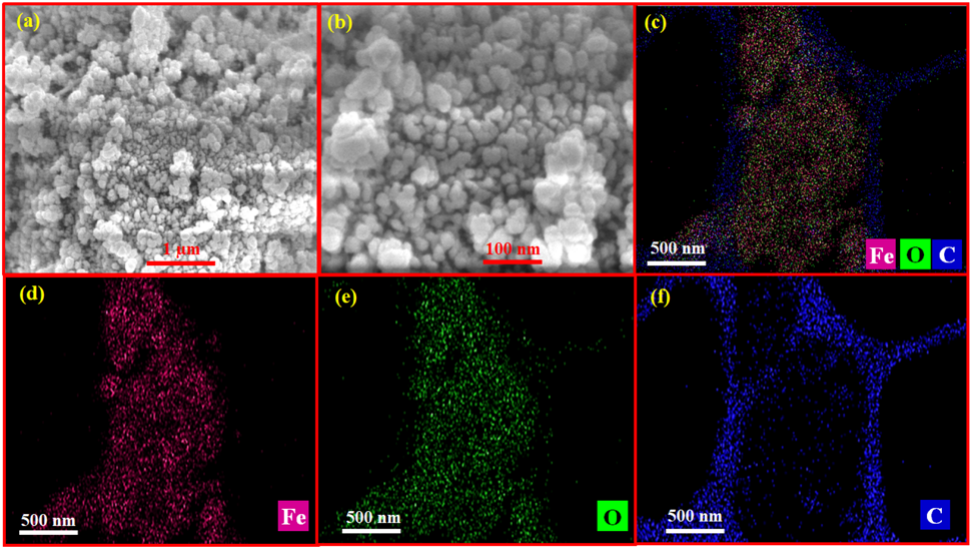
(a and b) FE-SEM images of synthesised Fe_3_O_4_@BTC NCs and representative elemental distribution images depicted in (c–f) for individual elements.

Field emission scanning electron microscope (FE-SEM) and elemental mapping (EDAX) images provides complementary insights about surface topography, particle size and shape of Fe_3_O_4_@BTC NCs. As shown in Fig. 3a and b. It was found that the particles have well distributed spherical morphology with smooth surface. Additionally, the chemical constituent of the Fe_3_O_4_ and Fe_3_O_4_@BTC NCs were indicated by EDAX analysis. The EDAX results of Fe_3_O_4_ and Fe_3_O_4_@BTC NC is depicted in S1 (Fig. 1f) and in S2 (Fig. 2). Moreover, the EDAX results of Fe_3_O_4_@BTC NCs, showed the presence of Fe, O and C elements with weight percentage of 41.4, 29.9, 28.8% respectively. Which strongly indicates the successfully formation of Fe_3_O_4_@BTC NCs. Furthermore, elemental mapping images unveil the homogenous distribution of Fe, O and C over the catalyst surface as shown in Fig. 3.

### High-resolution transmission electron microscopy (HR-TEM) analysis

**Fig. 4:**
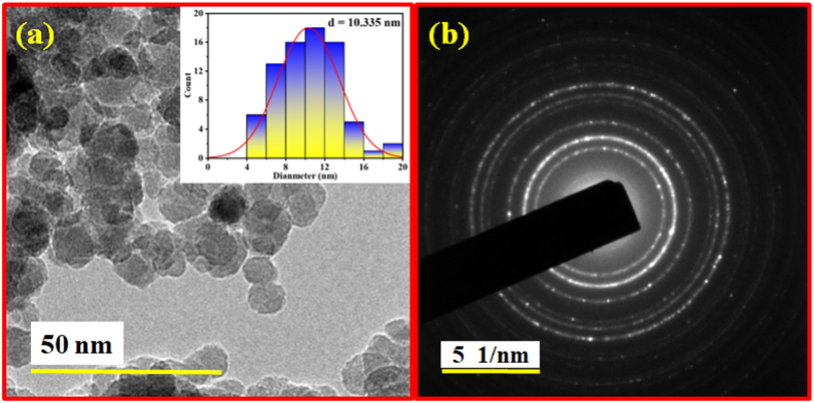
(a) High-resolution transmission electron microscopy (HR-TEM) images of Fe_3_O_4_@BTC NCs and inset of (a) shows the particle size distribution curve which shows the particle size is 10.335 nm, and (b) selective area diffraction (SAED) pattern of Fe_3_O_4_@BTC NCs.

In high-resolution transmission electron microscopy (HR-TEM) technique, information at atomic level of synthesised Fe_3_O_4_@BTC NCs could be obtained. HR-TEM images of synthesised Fe_3_O_4_@BTC were represented in Fig. 4. HR-TEM images of Fe_3_O_4_@BTC at different magnification predicts that the NCs had a spherical shape. In inset of (a) shows the particle size distribution curve which shows the particle size is ∼10.335 nm. The selective area diffraction (SAED) pattern of the synthesised NCs shows, it is polycrystalline in nature and is in good agreement with XRD analysis Fig. 1A.

### Catalytic functions of Fe_3_O_4_@BTC NCs

After synthesis and characterization of the Fe_3_O_4_@BTC NCs, the activity catalyst is evaluated in the synthesis of dihydropyrano[2,3-*c*]pyrazoles derivatives. In order to optimize protocol for synthesizing product *via* one pot MCR approach, model reaction was examined. This reaction involved benzaldehyde, ethyl acetoacetate, malononitrile and hydrazine hydrate with different catalyst concentrations under ultrasound irradiations. The effect of various reaction parameters such as catalyst loading, solvents, temperature, activation sources was extensively investigated (entries 1–15, Table 1). The results procured are illustrated in Table 1. Initially, desired product was obtained in the absences catalyst, solvent, at room temperature. It was found that final product was obtained in a trace amount within 10 min (entry 1). Before optimizing the amount of catalyst used, the model was performed under reflux and ultrasonic conditions. However, insufficient 25 and 30% yield was noticed respectively (entry 2 and 3). Consequently, efficiency of amount of catalyst on the rate of reaction was also investigated. The amount of catalyst has been varied as 0.01, 0.02, 0.03, 0.04 and 0.05 g (entry 4–8, Table 1) and results are illustrated in Table 1. According to these observations, the best result was obtained with 0.04 g of Fe_3_O_4_@BTC NCs at room temperature under ultrasonic irradiation (entry 7). Moreover, the solvent effect was also examined by using several solvents such as, H_2_O, DCM, CH_3_OH and C_2_H_5_OH (entry 9–12). The results show that C_2_H_5_OH can be a suitable solvent for reaction, which can provide 45% yield of product (entry 12). After optimizing reaction conditions, in order to study the role of respective components of catalyst model reaction have been repeated with Fe_3_O_4_, FeCl_2_ and BTC. The results are summarized in Table 1. The obtained results indicates that, initially Fe_3_O_4_ as a catalyst (entry-13, Table 1) with reaction time of 15 min at ultrasound irradiation under solvent free conditions yielded moderate amount (35% yield) of desired product. However the use of FeCl_3_ as a catalyst (entry-14 Table 1) in model reaction, yield of 30% was obtained within 20 min reaction treatment. Then, at the end, performing reaction in BTC (entry-15 Table 1) under ultrasound irradiation present the desired product in a 37% yield after 14 min. These results show high yield of desired dihydropyrano[2,3-*c*]pyrazoles product than Fe_3_O_4_ and FeCl_3_. The above findings reveals that in NCs the incorporation of BTC to Fe_3_O_4_ increases the acidic sites and thereby enhances the active sites of catalyst.

**Table 1:** Optimization of solvent and Fe_3_O_4_@BTC NCs loading in the synthesis of in dihydropyrano[2,3-*c*]pyrazoles derivatives^a^

Entry	Catalyst (g)	Solvent	Condition	Time (min)	Yield^b^ (%)
1	Without catalyst	—	RT	10	Trace
2	Without catalyst	—	Reflux	10	25
3	Without catalyst	—	U.S.(60 W) (RT)	10	30
4	Fe_3_O_4_@BTC (0.01)	—	U.S.(60 W) (RT)	10	50
5	Fe_3_O_4_@BTC (0.02)	—	U.S.(60 W) (RT)	10	75
6	Fe_3_O_4_@BTC (0.03)	—	U.S.(60 W) (RT)	10	80
7	Fe_3_O_4_@BTC (0.04)	—	U.S.(60 W) (RT)	10	92
8	Fe_3_O_4_@BTC (0.05)	—	U.S.(60 W) (RT)	10	92
9	Fe_3_O_4_@BTC (0.04)	H_2_O	U.S.(60 W) (RT)	20	30
10	Fe_3_O_4_@BTC (0.04)	DCM	U.S.(60 W) (RT)	10	40
11	Fe_3_O_4_@BTC (0.04)	CH_3_OH	U.S.(60 W) (RT)	15	41
12	Fe_3_O_4_@BTC (0.04)	C_2_H_5_OH	U.S.(60 W) (RT)	7	45
13	Fe_3_O_4_ (0.04)	—	U.S.(60 W) (RT)	15	35
14	FeCl_2_ (0.04)	—	U.S.(60 W) (RT)	20	30
15	BTC (0.04)	—	U.S.(60 W) (RT)	14	37

a RT – room temperature; reaction conditions: benzaldehyde (1 mmol), hydrazine hydrate (1 mmol), ethyl acetoacetate (1 mmol), malononitrile (1 mmol), ultra-sonication (60 W).

b Isolated yield.

Therefore, benzaldehyde (1 mmol), ethyl acetoacetate (1 mmol), malononitrile (1 mmol) and hydrazine hydrate (1 mmol), 0.04 g of Fe_3_O_4_@BTC NCs, solvent free, under ultrasound irradiation were selected as best reaction conditions.

After optimizing different parameters, to evaluate scope of these method, various derivatives of dihydropyrano[2,3-C]pyrazoles were synthesised by reacting various aldehydes. These results were summarized in (entries 1–11, Table 2). These products structures were characterized by using ^1^H NMR, ^13^C NMR, FT-IR, ESI-MS spectra and melting points, added in SI (S3, S4 and S5).

**Table 2:** Synthesis of dihydropyrano[2,3-*c*]pyrazole derivatives in the presence of Fe_3_O_4_@BTC NCs

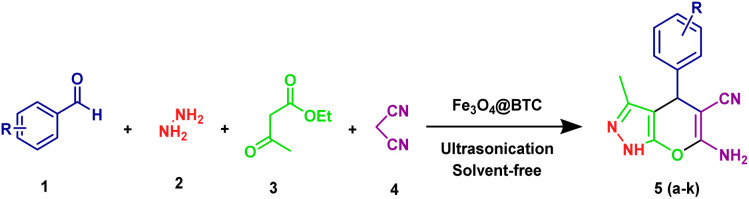						
Entry	Aldehyde	Product	Time (min)	Yield (%)	MP (°C) found	MP (°C) reported
1	C_6_H_5_CHO	5a	7	92	240–242	241–243 (ref. 29)
2	2-NO_2_–C_6_H_4_CHO	5b	5	89	220–222	223–225 (ref. 28)
3	3-NO_2_–C_6_H_4_CHO	5c	5	89	237–239	239–242 (ref. 59)
4	4-NO_2_–C_6_H_4_CHO	5d	4	90	192–194	191–193 (ref. 29)
5	2-Cl–C_6_H_4_CHO	5e	6	85	260–262	267–268 (ref. 60)
6	4-Cl–C_6_H_4_CHO	5f	5	91	225–227	230–232 (ref. 28)
7	2-OH–C_6_H_4_CHO	5g	6	90	209–211	210–212 (ref. 61)
8	3-OH–C_6_H_4_CHO	5h	8	87	255–257	259–261 (ref. 61)
9	4-OH–C_6_H_4_CHO	5i	10	79	210–213	215 (ref. 28)
10	4-OCH_3_–C_6_H_4_CHO	5j	12	80	205–207	206–208 (ref. 29)
11	4-CH_3_–C_6_H_4_CHO	5k	7	85	170–174	174–176 (ref. 29)

### Catalyst recyclability

In the direction of investigating recyclability study of Fe_3_O_4_@BTC NCs was monitored using model reaction with optimized reaction conditions (Scheme 1). After the completion of reaction, the reaction mixture was dissolved in ethanol and then the NCs was separated by using an external magnet.

**Fig. 5:**
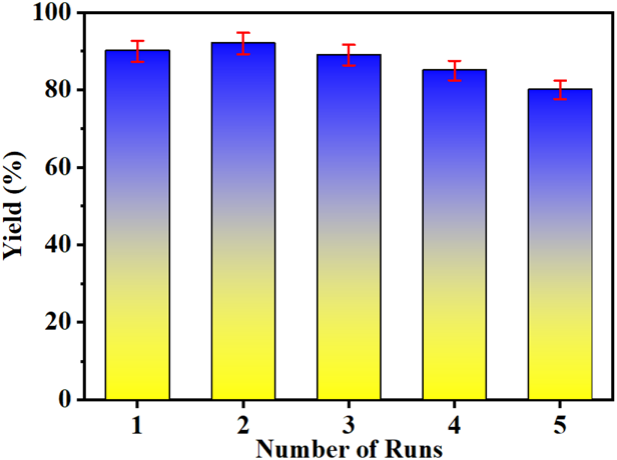
Recyclability of Fe_3_O_4_@BTC NCs after 5 consecutive runs.

**Fig. 6:**
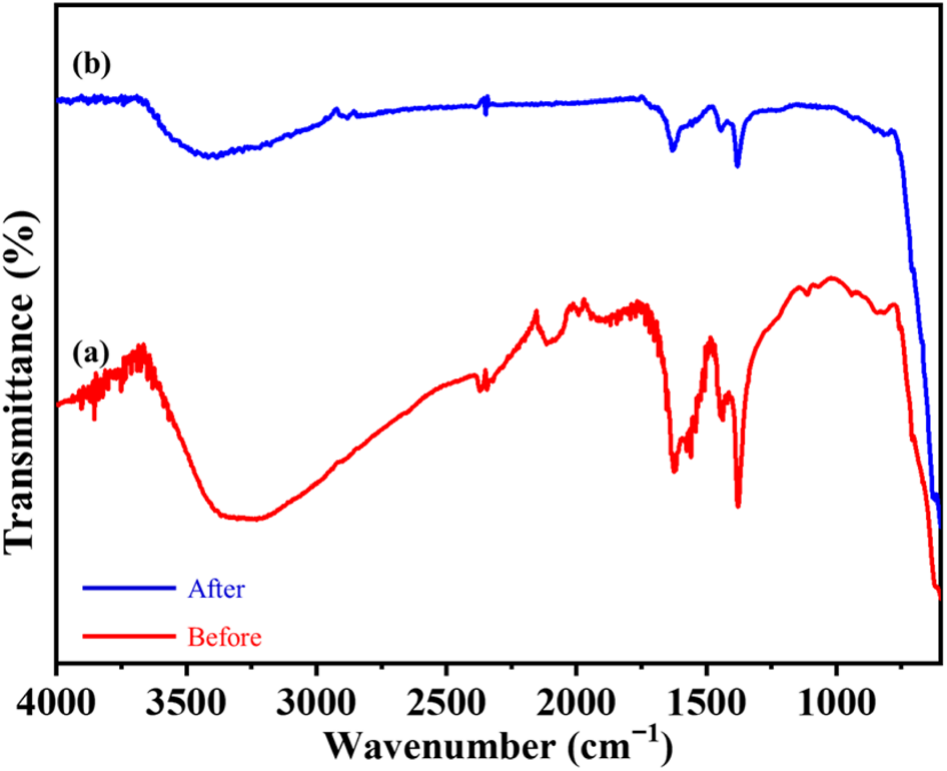
Superimposed FT-IR spectra of (a) fresh and (b) recovered Fe_3_O_4_@BTC NCs (after catalytic studies).

The reacquired NCs was washed several times with ethanol, dried at 80 °C in oven and reused for next run. The reacquired NCs can be used up to five times with no significant loss of catalytic activity shown in Fig. 5. The FT-IR of the catalyst after five cycles was recorded and does not show any considerable change compared to the fresh catalyst, as shown in Fig. 6. Which is in the support during recycling process sustained unchanged. However, there is slight decrease in yield from 92 to 84% has been noticed. The reaction mixture was ultrasonicated at 25 °C and after completion of reaction, temperature is about 29 °C. It can be clearly seen that yield has decreased by about 8%, might be due to under ultrasonic conditions which leads to partial deactivation of catalyst and few of reactive sites are inactive. In the progress of reaction catalyst loading was diminished between each cycle may be responsible to diminished the reaction yield.

### Comparison of the catalyst

In order to show competences and efficiency of Fe_3_O_4_@BTC NCs was determined comparatively with some previously reported methods for the synthesis of dihydropyrano[2,3-*c*]pyrazoles. The observations depicted in Table 3, show that the Fe_3_O_4_@BTC NCs show better results over reported catalyst. As indicated in Table 3, NCs used in the this MCR reaction shows characteristic features like short reaction time, high yield, nontoxic, economical, easy workup and recyclability.

**Table 3:** Comparison of synthesised Fe_3_O_4_@BTC as catalyst for the synthesis of dihydropyrano[2,3-*c*]pyrazoles derivatives with other catalyst

Entry	Catalyst	Condition	Time (min)	Yield (%)	Ref.
1	PdO/Al-SBA-15	H_2_O/EtOH, reflux	20	85	62
2	ZnO@PEG, EtOH	Ultrasonication	15	87	63
3	Biochar-Fe_3_O_4_–TiO_2_	EtOH : H2O, 60 °C	10	91	64
4	Y_3_Fe_5_O_12_	Solvent free, 80 °C	20	89	65
5	K09(natural phosphate)	Ethanol, RT	20	85	66
6	γ-Alumina	H_2_O/reflux	35	90	67
7	Bovine serum albumin (BSA)	90% aq. EtOH	70	95	68
**8**	**Fe**_**3**_ **O**_**4**_ **@BTC**	**Solvent free, sonication**	**7**	**92**	**This work**

### Proposed mechanism

**Scheme 4:**
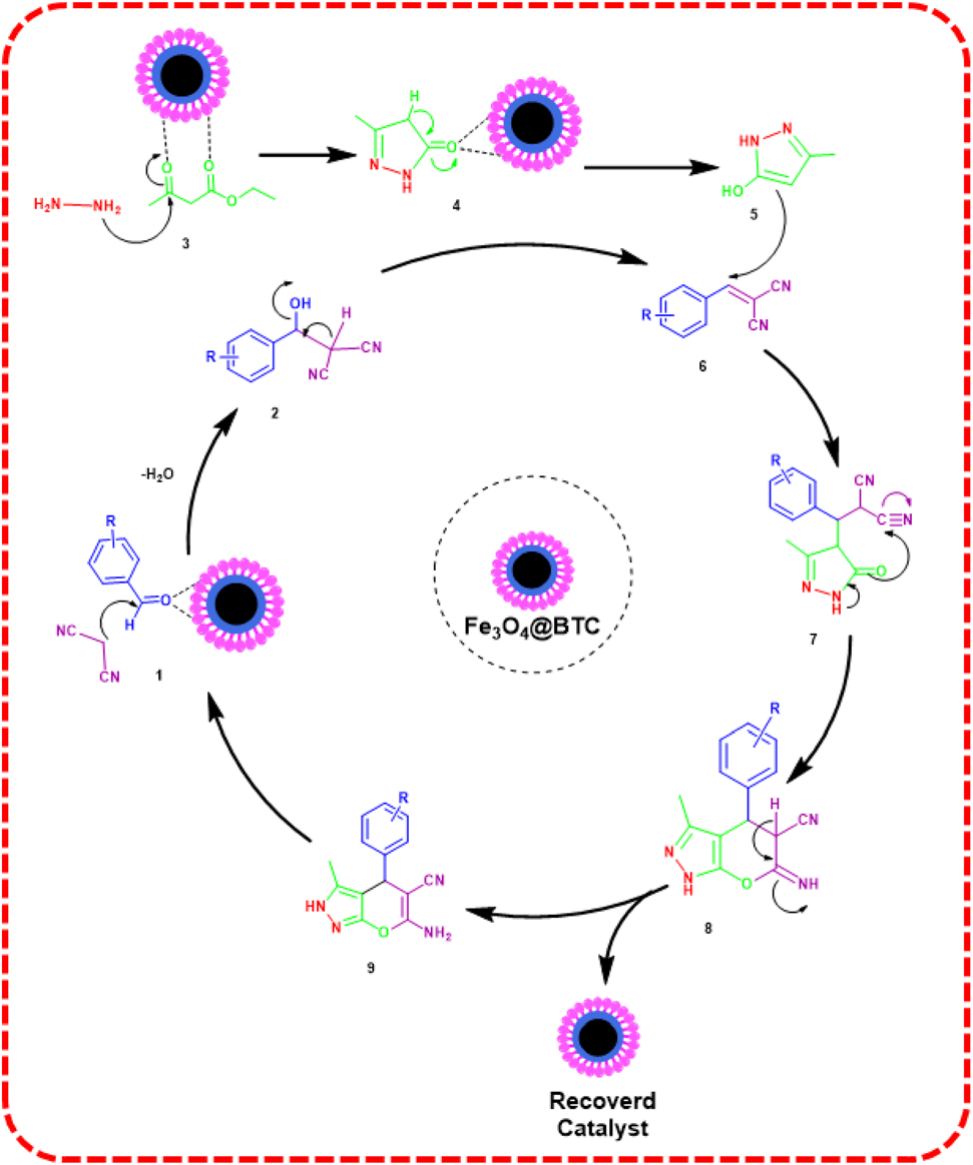
Proposed mechanism for the synthesis of dihydropyrano[2,3-*c*]pyrazoles and its derivatives in the presence of Fe_3_O_4_@BTC NC.

The plausible mechanism for the synthesis of dihydropyrano[2,3-*c*]pyrazoles derivatives using Fe_3_O_4_@BTC NCs has been shown in Scheme 4. As can be seen, NCs activate the carbonyl group of ethyl acetoacetate. In the first step, involves nucleophilic attack of –NH_2_ groups of hydrazine hydrate on carbonyl group of ethyl acetoacetate. Here, losing of H_2_O, which leads to formation of intermediate pyrazolone (5).^69^ In the next step, NCs activates the C=O functional group of aldehydes and facilitates Knoevenagel condensation with malononitrile, results in the formation of the intermediate (6). Then, Michael addition reaction between intermediate (5) and (6) resulted in intermediate (7), which goes through intramolecular cyclization, providing intermediate (8). Finally, through tautomerization of intermediate (8), the desired dihydropyrano[2,3-*c*]pyrazole (9) was obtained.^28^

## Conclusions

In summary, an efficient, and environmentally friendly heterogeneous nanocatalyst Fe_3_O_4_@BTC has been successfully synthesised. The synthesised NCs was characterized using various characterization techniques such as FT-IR, FE-SEM, HR-TEM, EDAX, BET and TGA analysis. The FE-SEM and HR-TEM results confirms it is ∼13 nm in size, uniform distribution of elements and it has spherical shape. The TGA analyses confirmed high thermal and chemical stability. BET surface area analysis proclaims its surface area 84.87 m^2^ g^−1^ resulting into enhanced catalytic activity. This NCs proved to be an efficient catalyst for the one pot synthesis of dihydropyrano[2,3-*c*] pyrazoles derivatives under solvent free conditions with excellent yields. Moreover, NCs simply recovered and it can be reused for five consecutive cycles without any notable loss in its catalytic activity. A complete structural analysis of synthesised derivatives was confirmed using FT-IR, ^1^H NMR, ^13^C NMR and ESI-MS. This protocol provides several advantages, which include efficient, economical, low catalyst loading, high yield, short reaction time, simple workup, solvents free conditions and recyclability of catalyst.

## Author contributions

Santosh A. Fuse: writing – original draft, validation, methodology, investigation, formal analysis, data curation. Somnath C. Dhawale: writing – review & editing, validation, methodology, formal analysis, data curation. Balaji B. Mulik: validation, methodology, investigation. Raviraj P. Dighole: review & editing, validation, software, investigation. Balaji R. Madje: review & editing, supervision, validation, software, investigation, formal analysis. Bhaskar R. Sathe: writing – review & editing, visualization, validation, supervision, software, resources, project administration, methodology, investigation, funding acquisition, formal analysis, conceptualization.

## Conflicts of interest

The authors declare no conflict of interest.

## Supplementary Material

RA-015-D5RA08120C-s001

## Data Availability

The data supporting this article have been included as part of the supplementary information (SI). Supplementary information: (a) SEM, EDAXS and elemental mapping images of Fe_3_O_4_, (b) DAXS of Fe_3_O_4_@Fe-BTC NC. (c) FT-IR, ^1^H NMR, ^13^C NMR and mass spectra of (5a): 6-amino-1,4-dihydro-3-methyl-4-phenylpyrano[2,3-*c*]pyrazole-5-carbonitrile. (d) FT-IR, ^1^H NMR, and ^13^C NMR (5j): 6-amino-4-(2-chlorophenyl)-3-methyl-1,4-dihydropyrano[2,3-*c*]pyrazole-5-carbonitrile. (e) FT-IR, ^1^H NMR, and ^13^C NMR (5j): 6-amino-4-(4-methoxyphenyl)-3-methyl-1,4-dihydropyrano[2,3-*c*]pyrazole-5-carbonitrile. See DOI: https://doi.org/10.1039/d5ra08120c.
