# Supplementary material for: Magnetic Fe3O4@BTC nanocomposite for ultrasound-assisted synthesis of dihydropyrano[2,3-c]pyrazoles
Source: RSC Adv. 2025 Dec 12;15(58):49669–77. doi: 10.1039/d5ra08120c (PMC12699521; doi:10.1039/d5ra08120c)
Supplement: RA-015-D5RA08120C-s001 [file RA-015-D5RA08120C-s001.pdf]

Supplementary Information (SI) for RSC Advances.
This journal is © The Royal Society of Chemistry 2025

Supporting Information

Magnetic Fe3O4@BTC nanocomposite for ultrasound-assisted synthesis of dihydropyrano[2,3-c]pyrazoles

Santosh A. Fuse,[a, b, c] Somnath C. Dhawale,[a] Balaji B. Mulik,[a, d] Raviraj P. Dighole,[a, c] Balaji R. Madje[b]* and Bhaskar R. Sathe,[a, e]*

[a]Department of Chemistry, Dr. Babasaheb Ambedkar Marathwada University, Chhatrapati Sambhajinagar, Maharashtra 431004, India

[b]Vasantrao Naik College, Chhatrapati Sambhajinagar, Maharashtra 431003, India

[c]A. S. C. College, Badnapur, Dist.-Jalna, Maharashtra 431202, India

[d]MGM University, Chhatrapati Sambhajinagar, Maharashtra 431001, India

[e]Department of Nanoscience and Technology, Dr. Babasaheb Ambedkar Marathwada University, Chhatrapati Sambhajinagar, Maharashtra 431004, India

E-mail: *bsathe.chemistry@bamu.ac.in*

List of Supporting Information

S.1. SEM, EDAXS and elemental mapping images of Fe_3O_4.

S.2. EDAXS of Fe_3O_4@Fe-BTC NC.

S.3. FT-IR, ^{1}H NMR, ^{13}C NMR and Mass Spectra of (5a): 6-amino-1, 4-dihydro-3-methyl-4-phenylpyrano [2, 3-c] pyrazole-5-carbonitrile

S.4. FT-IR, ^{1}H NMR, and ^{13}C NMR (5j): 6-amino-4-(2-chlorophenyl)-3-methyl-1,4-dihydropyrano[2,3-c]pyrazole-5-carbonitrile.

S.5. FT-IR, ^{1}H NMR, and ^{13}C NMR (5j): 6-amino-4-(4-methoxyphenyl)-3-methyl-1,4-dihydropyrano[2,3-c]pyrazole-5-carbonitrile

S1. SEM, EDAX and elemental mapping images of Fe_3O_4

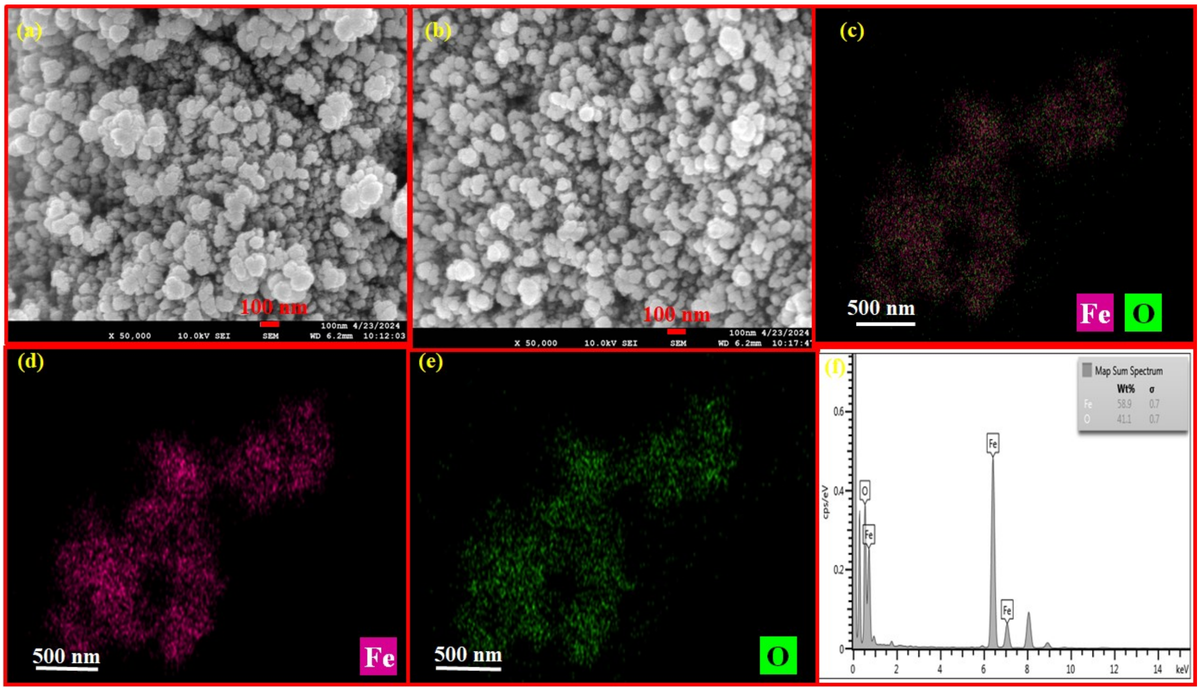

Figure 1: (a, b) SEM images, and (c-e) elemental mapping of Fe_3O_4 NPs, (f) EDAX images of Fe and O of Fe_3O_4 NPs.

S2. EDAX of Fe_3O_4@Fe-BTC NC

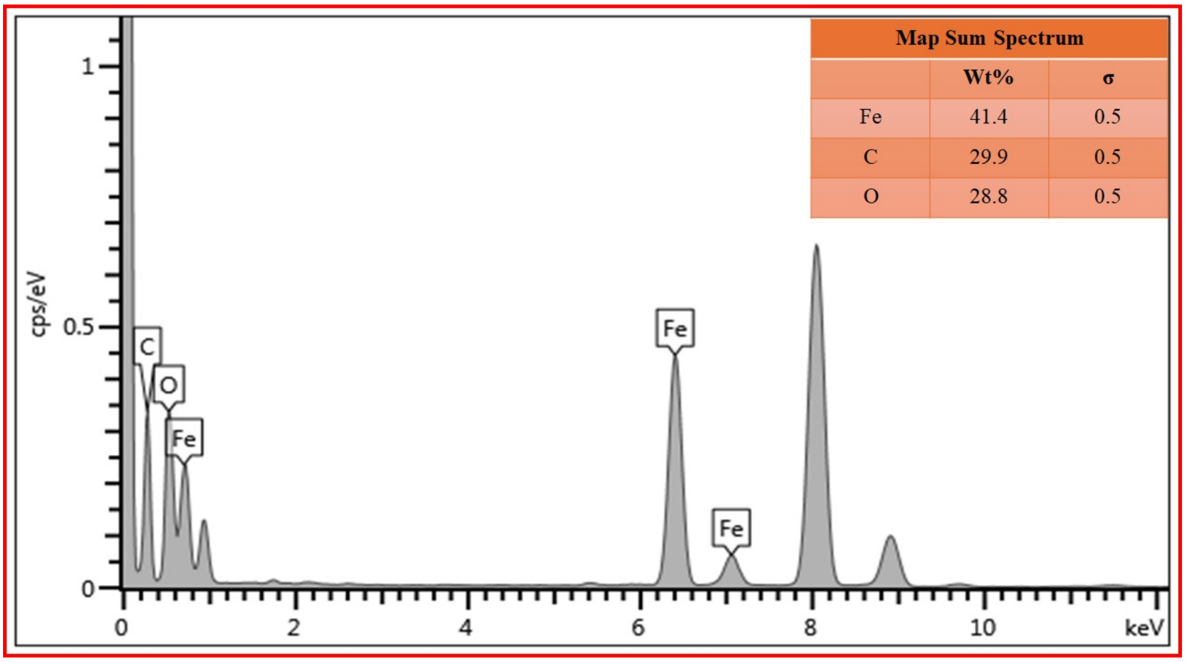

Figure 2: EDAX of Fe_3O_4@Fe-BTC NC.

S3: FT-IR, ¹H NMR, ¹³C NMR and mass spectra of (5a): 6-amino-1, 4-dihydro-3-methyl-4-phenylpyrano [2, 3-c] pyrazole-5-carbonitrile.

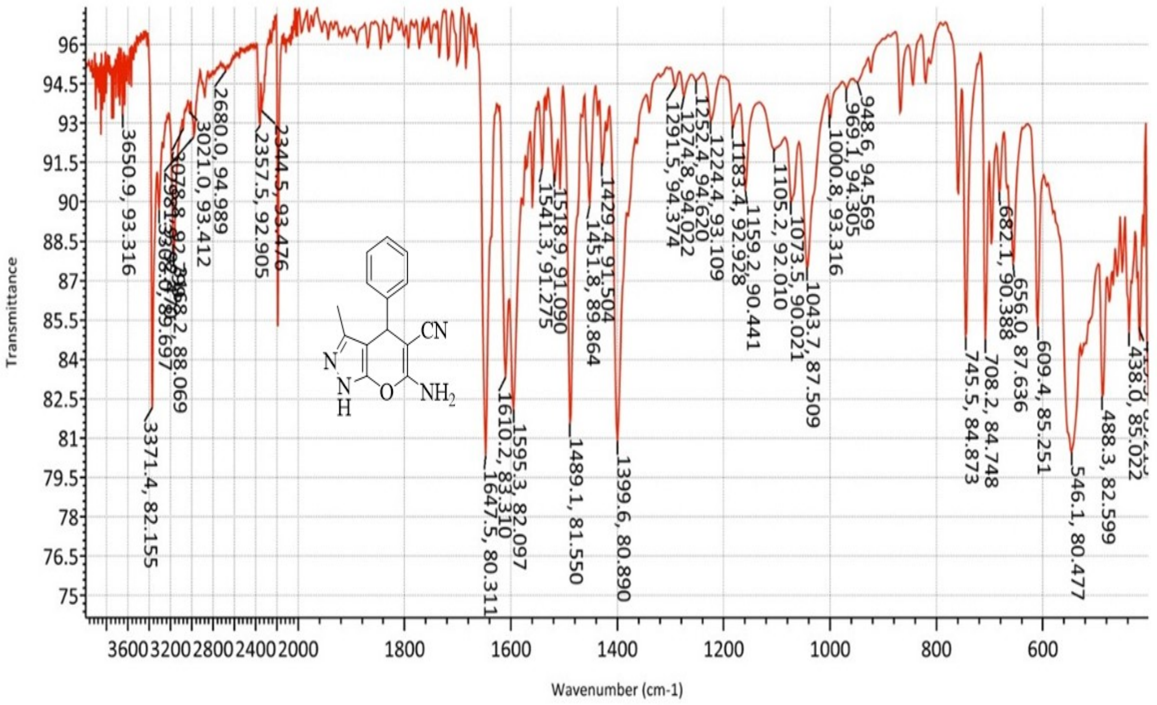

Figure 3: (5a) FT-IR of 6-amino-1, 4-dihydro-3-methyl-4-phenylpyrano [2, 3-c] pyrazole-5-carbonitrile

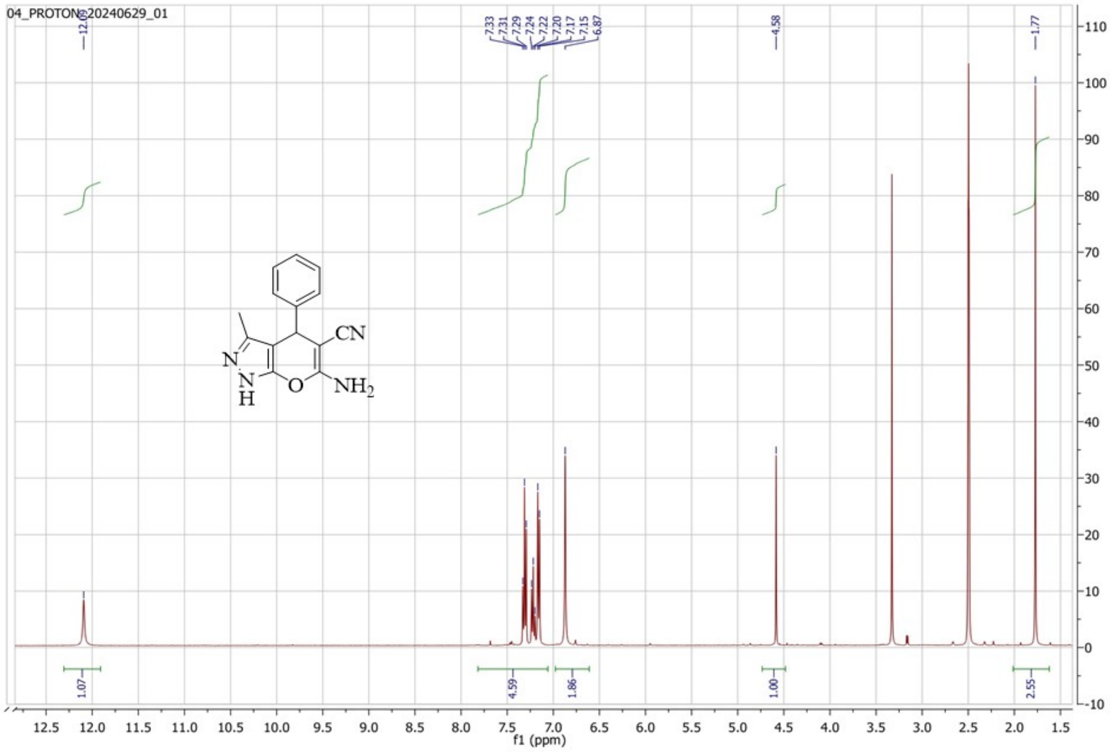

Figure 4: (5a) ^{1}H NMR of 6-amino-1, 4-dihydro-3-methyl-4-phenylpyrano [2, 3-c] pyrazole-5-carbonitrile.

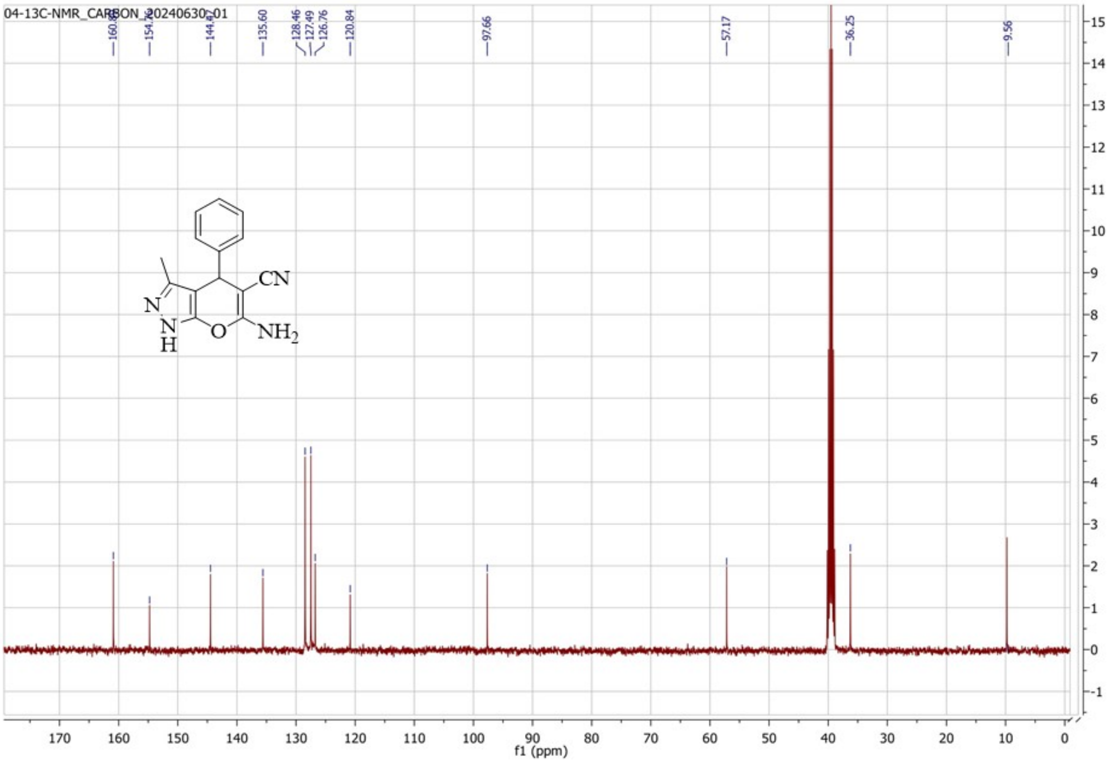

Figure 5: (5a) ^{13}C NMR of 6-amino-1, 4-dihydro-3-methyl-4-phenylpyrano [2, 3-c] pyrazole-5-carbonitrile.

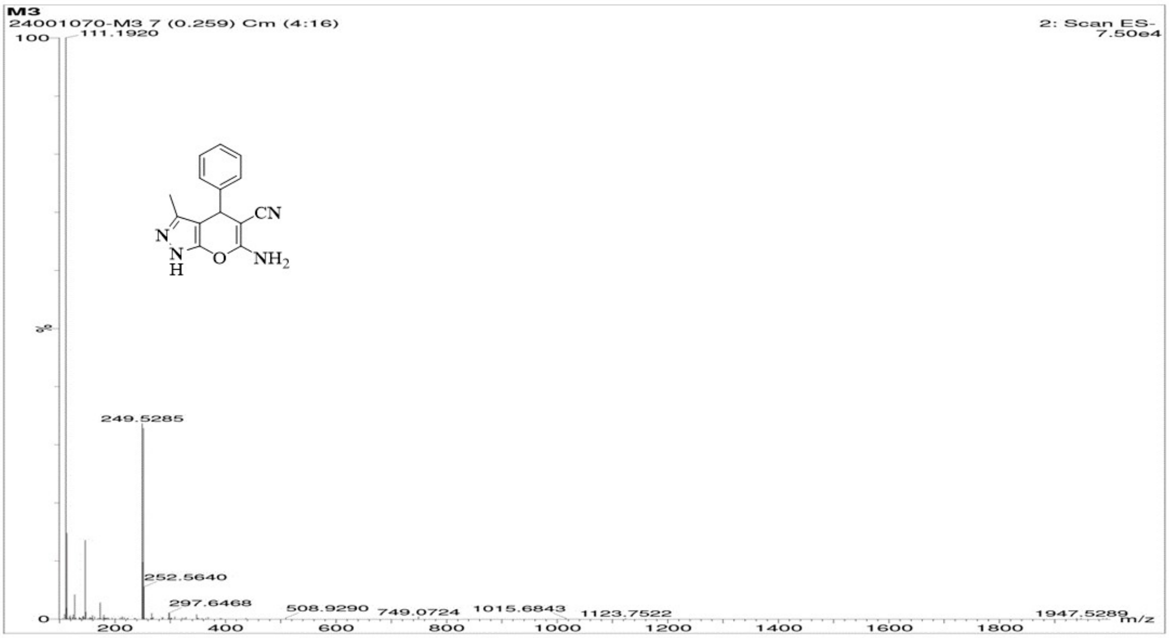

Figure 6: (5a) EIS-Mass of 6-amino-1, 4-dihydro-3-methyl-4-phenylpyrano [2, 3-c] pyrazole-5-carbonitrile

S2. FT-IR, ^{1}H NMR, and ^{13}C NMR of (5j): 6-amino-4-(2-chlorophenyl)-3-methyl-1,4-dihydropyrano[2,3-c]pyrazole-5-carbonitrile.

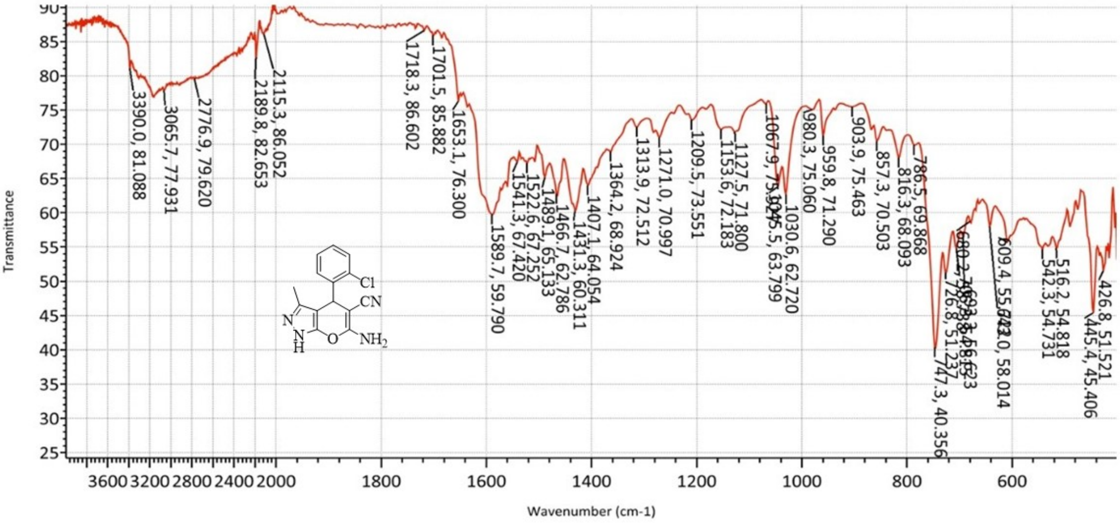

Figure 7: (5e) FT-IR of 6-amino-4-(2-chlorophenyl)-3-methyl-1,4-dihydropyrano[2,3-c] pyrazole-5-carbonitrile.

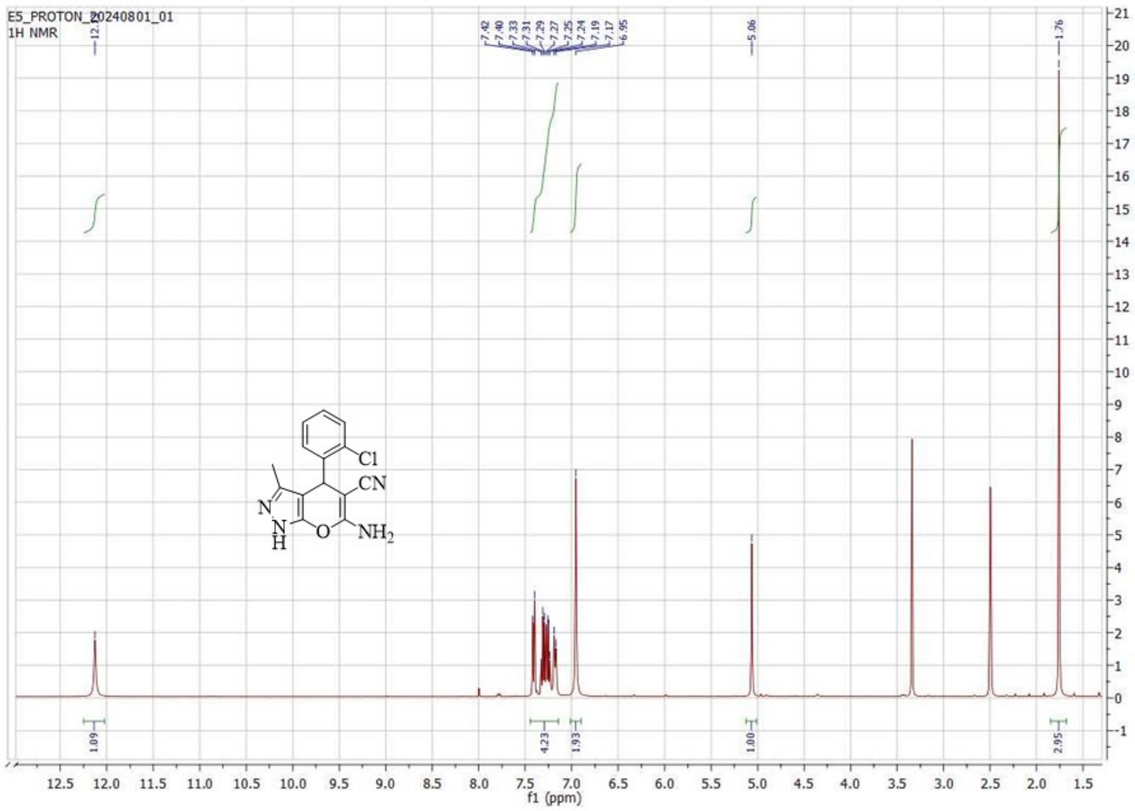

Figure 8: (5e) ^{1}H-NMR of 6-amino-4-(2-chlorophenyl)-3-methyl-1,4-dihydropyrano[2,3-c]pyrazole-5-carbonitrile

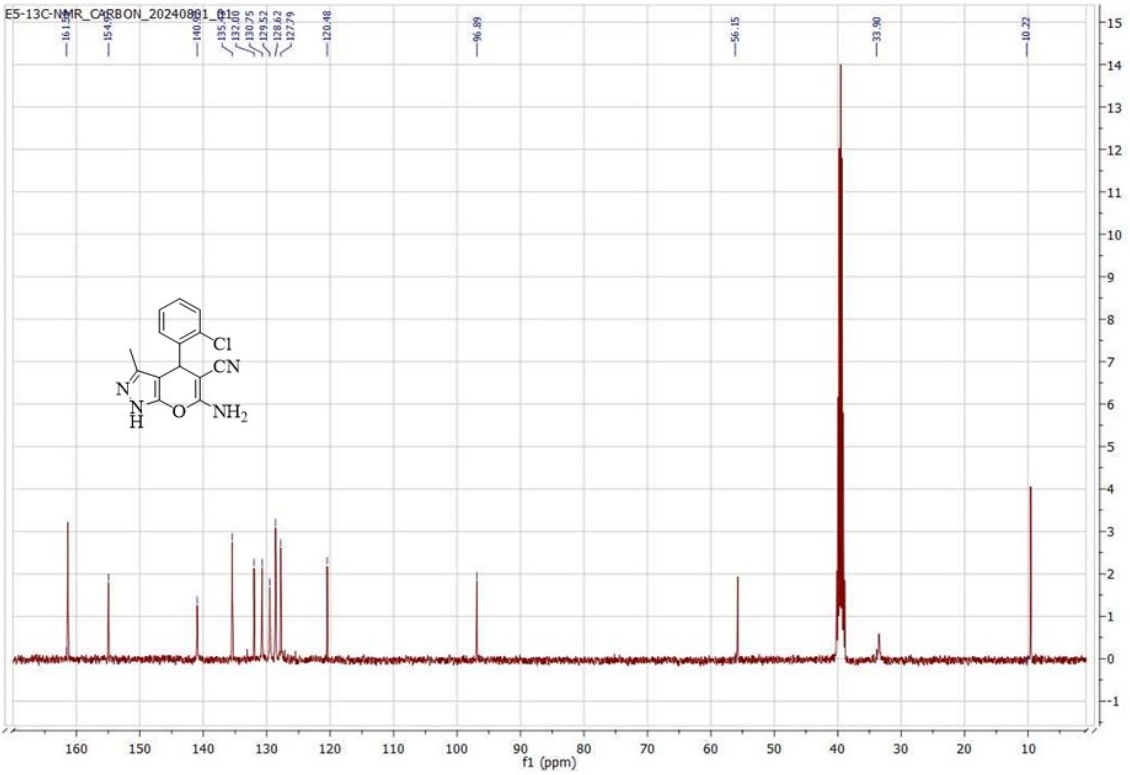

Figure 9: (5e) ^{13}C-NMR of 6-amino-4-(2-chlorophenyl)-3-methyl-1,4-dihydropyrano[2,3-c]pyrazole-5-carbonitrile.

S3. FT-IR, ^{1}H NMR, and ^{13}C NMR of (5j): 6-amino-4-(4-methoxyphenyl)-3-methyl-1,4-dihydropyrano[2,3-c]pyrazole-5-carbonitrile.

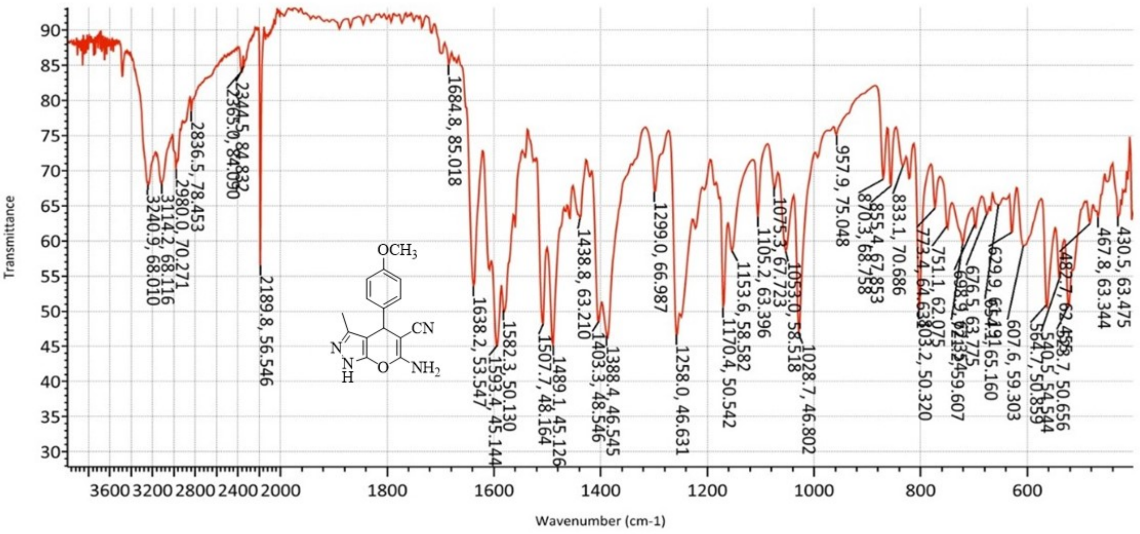

Figure 10: (5j) FT-IR of 6-amino-4-(4-methoxyphenyl)-3-methyl-1,4-dihydropyrano[2,3-c]pyrazole-5-carbonitrile

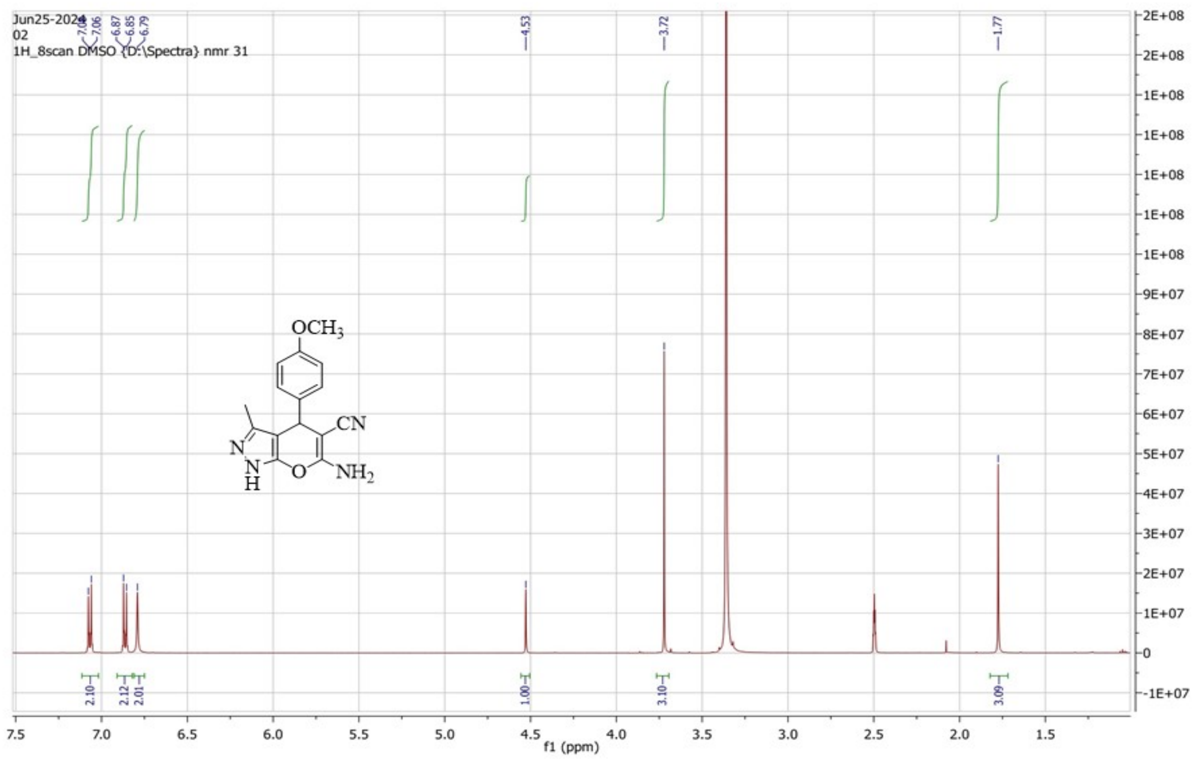

Figure 11: (5j) ^{1}H NMR of 6-amino-4-(4-methoxyphenyl)-3-methyl-1,4-dihydropyrano[2,3-c]pyrazole-5-carbonitrile

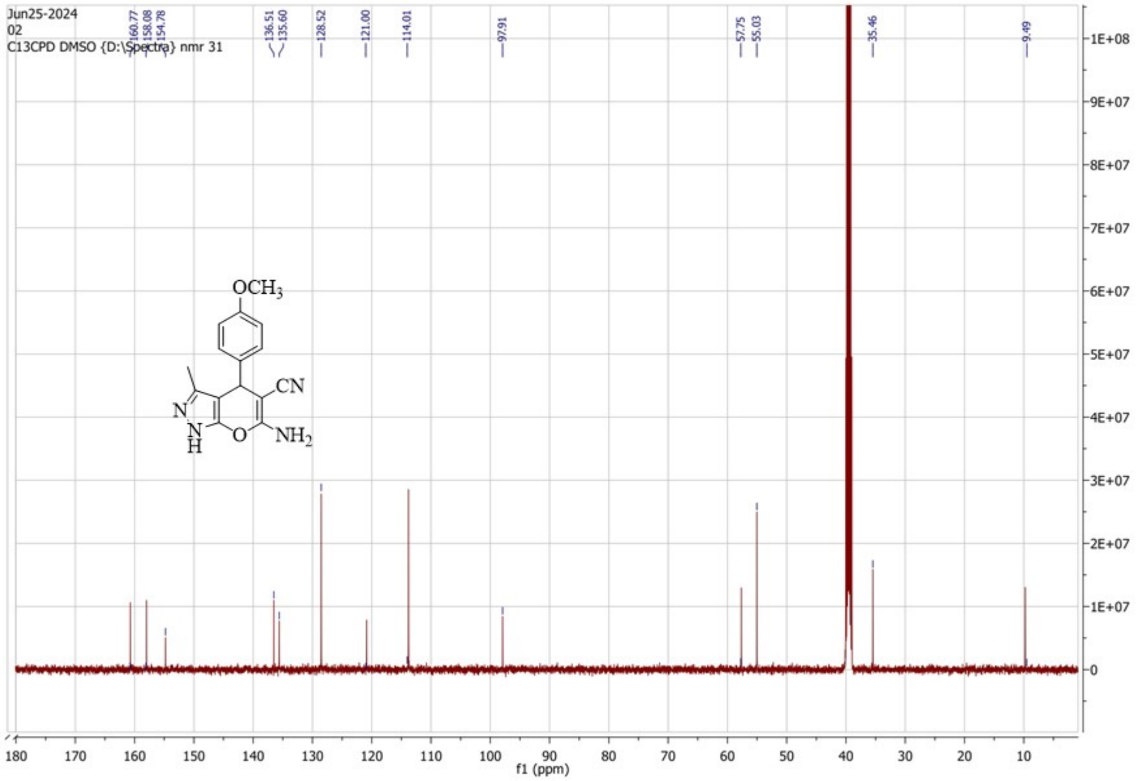

Figure 12: (5j) ^{13}C NMR of 6-amino-4-(4-methoxyphenyl)-3-methyl-1,4-dihydropyrano[2,3-c]pyrazole-5-carbonitrile.
